# Bathtub Curve Patterns of Mortality Risk in Cardiometabolic Diseases: A Cohort Study Using the UK Biobank

**DOI:** 10.1093/geroni/igaf122.2889

**Published:** 2025-12-31

**Authors:** Yuming Chen, Shui-kit Cheuk, Hui Liu, Yanran Deng, Beibei Xu

**Affiliations:** Peking University Health Science Center, Beijing, Beijing, China (People’s Republic); Peking University, Beijing, Beijing, China (People’s Republic); Peking University, Beijing, Beijing, China (People’s Republic); Peking University Health Science Center, Beijing, Beijing, China (People’s Republic); Peking University, Beijing, Beijing, China (People’s Republic)

## Abstract

Observational studies suggest nonlinear mortality patterns in cardiometabolic diseases (CMDs): acute events show early risk spikes, while chronic conditions exhibit gradual risk accumulation. These trends superficially align with the bathtub curve—an engineering model describing triphasic failure rates. However, systematic validation of this framework in chronic disease epidemiology remains limited. This study aims to explore and validate the nonlinear temporal dynamics of mortality risk in CMD patients based on bathtub curve theory. Using data from the UK Biobank (n = 502,130), we analyzed 17 CMDs with linked primary care, inpatient and death records among 73,908 patients. A piecewise exponential survival model segmented mortality risk into intervals to capture time-dependent hazard rates. Chronic (e.g., diabetes) and acute diseases (e.g., myocardial infarction) were analyzed separately. Models were adjusted for age, sex, BMI, education, and smoking/alcohol status. Chronic diseases, such as diabetes, exhibited a bathtub-shaped mortality risk: elevated initially, declining in mid-term, and rising again long-term. Each phase showed increased risk with older age at diagnosis. Acute conditions (e.g., heart failure) demonstrated rapidly declining risks over time. Heterogeneity in high-risk time windows was observed across diseases, with hypertension and diabetes consistently following the bathtub curve. Using the piecewise exponential model, we validate the bathtub curve framework could provide a novel nonlinear perspective for understanding mortality risk evolution in CMDs. This approach supports stratified patient management, intensive early intervention, stable-phase monitoring, and late-phase complication control, enhancing precision in public health strategies. The findings underscore the theory’s potential in optimizing lifecycle management of CMDs and guiding personalized interventions.

